# p53 cooperates with SIRT6 to regulate cardiolipin de novo biosynthesis

**DOI:** 10.1038/s41419-018-0984-0

**Published:** 2018-09-20

**Authors:** Meiting Li, Tianyun Hou, Tian Gao, Xiaopeng Lu, Qiaoyan Yang, Qian Zhu, Zhiming Li, Chaohua Liu, Guanqun Mu, Ge Liu, Yantao Bao, He Wen, Lina Wang, Haiying Wang, Ying Zhao, Wei Gu, Yang Yang, Wei-Guo Zhu

**Affiliations:** 1 Key Laboratory of Carcinogenesis and Translational Research (Ministry of Education), Beijing Key Laboratory of Protein Posttranslational Modifications and Cell Function, Department of Biochemistry and Molecular Biology, School of Basic Medical Sciences, Peking University Health Science Center, 38 Xueyuan Road, Beijing, 100191 China; 2 Guangdong Key Laboratory for Genome Stability and Human Disease Prevention, Carson cancer research center, Department of Biochemistry and Molecular Biology, School of Medicine, Shenzhen University, Shenzhen, 516080 China; 3 Institute for Cancer Genetics, Department of Pathology and Cell Biology, Herbert Irving Comprehensive Cancer Center, College of Physicians & Surgeons, Columbia University, 1130 Nicholas Ave, New York, NY 10032 USA

## Abstract

The tumor suppressor p53 has critical roles in regulating lipid metabolism, but whether and how p53 regulates cardiolipin (CL) de novo biosynthesis is unknown. Here, we report that p53 physically interacts with histone deacetylase SIRT6 in vitro and in vivo, and this interaction increases following palmitic acid (PA) treatment. In response to PA, p53 and SIRT6 localize to chromatin in a p53-dependent manner. Chromatin p53 and SIRT6 bind the promoters of CDP-diacylglycerol synthase 1 and 2 (*CDS1* and *CDS2*), two enzymes required to catalyze CL de novo biosynthesis. Here, SIRT6 serves as a co-activator of p53 and effectively recruits RNA polymerase II to the *CDS1* and *CDS2* promoters to enhance CL de novo biosynthesis. Our findings reveal a novel, cooperative model executed by p53 and SIRT6 to maintain lipid homeostasis.

## Introduction

The tumor suppressor p53 is known as the “guardian of the genome” due to its role in maintaining normal cell growth and genomic stability via cell-cycle regulation and inducing apoptosis and DNA damage repair in response to cellular stress^1,2^. Recent studies, however, have suggested that p53 may exert much broader cellular functions, including regulating lipid metabolism. Overexpression of wild-type p53 upregulates Caveolin 1 and consequently decreases intracellular-free cholesterol in human skin fibroblasts^3^. In addition, p53 can repress lipid anabolism by inhibiting the expression of sterol regulatory element-binding proteins 1c (*SREBP1c*) and its target genes^4^. Wild-type p53 also reduces nicotinamide adenine dinucleotide phosphate (NADPH) production and inhibits lipid accumulation by directly binding to glucose-6-phosphate dehydrogenase (G6PD) in colorectal carcinoma cells^5^. Finally, p53-mediated transcriptional induction of lipin 1 (Lpin1) following a 24 h starvation increases fatty acid oxidation (FAO) in the mouse liver^6^, which indicates a role for p53 in enhancing fatty acid catabolism. Functional studies of p53 in other modes of lipid metabolism, including lipid droplet accumulation^7^ and ceramides^8^ have also been performed, and have confirmed that p53 has an important role in regulating lipid metabolism.

Sirtuin 6 (SIRT6) is a member of the mammalian sirtuins family, and is another important molecule involved in lipid metabolism. SIRT6 is a chromatin regulatory protein and a critical mediator of diverse cellular processes^9–12^. SIRT6-overexpressing mice fed a high-fat diet show decreased visceral fat accumulation, and an improved blood lipid profile, glucose tolerance and insulin secretion, indicating that SIRT6 can dramatically affect lipid homeostasis^13^. Conversely, SIRT6 deficiency increases the expression of genes responsible for hepatic long-chain fatty-acid uptake and triglyceride synthesis, and reduces the expression of genes required for β-oxidation^14,15^. Sirt6 is also a critical mediator of low-density lipoprotein (LDL)-cholesterol homeostasis via control of proprotein convertase subtilisin/kexin type 9 (*Pcsk9*) gene expression^16^. Despite these cumulative data, the challenge remains to explore whether SIRT6 influences other forms of lipid metabolism.

Cardiolipin (CL) is a mitochondria-specific phospholipid involved in many important physiological functions, including the mitochondrial respiratory chain and oxidative phosphorylation (OXPHOS), mitochondria protein import, mitochondrial membrane stability and dynamics, and apoptosis^17–20^. In human cells, CL de novo biosynthesis is catalyzed first by CDP-diacylglycerol synthase (CDS) enzymes CDS1 and CDS2^21^, followed by phosphatidylglycerophosphate synthase 1 (PGS1)^22^, then protein-tyrosine phosphatase, mitochondrial 1 (PTPMT1)^23^, and finally CL synthase (CRLS1)^24^. CL is the signature mitochondrial phospholipid, and disturbances in CL de novo biosynthesis or CL molecular species composition have been associated with mitochondrial dysfunction in several pathophysiological conditions and diseases, such as Barth syndrome, diabetes, heart failure, and aging^25^.

Palmitic acid (PA) can be synthesized de novo, and is the most common saturated long-chain fatty acid found in food. PA has an important role in glucose and lipid metabolism, and PA overloading can induce metabolic disorders. PA may also induce lipid accumulation^26,27^ and atherosclerosis^28^ and is positively associated with incident type 2 diabetes^29^. Consequently, PA is usually used to investigate factors involved in lipid metabolism.

Our previous study reported that p53 activates *SIRT6* expression by binding the *SIRT6* promoter to regulate gluconeogenesis^30^. Here, we were interested to determine whether p53 and SIRT6 work together to regulate CL de novo biosynthesis. Consistent with previous studies^31^, we first demonstrated that p53 directly interacts with SIRT6. In addition, we observed that the complex recruits SIRT6 to chromatin upon PA exposure. Both p53 and SIRT6 bind the *CDS1/CDS2* promoters, which are key enzymes for CL de novo biosynthesis. SIRT6 effectively recruits RNA polymerase II to the *CDS1/CDS2* promoters to co-activate *CDS1* and *CDS2* expression. Altogether, our data demonstrate that both p53 and SIRT6 have a major role in regulating CL de novo biosynthesis. These findings further our understanding of the effects of p53 and SIRT6 on lipid metabolism and may guide the design of new therapeutics to regulate lipid homeostasis.

## Results

### p53 and SIRT6 expression increases after palmitic acid (PA) treatment

**Fig. 1:**
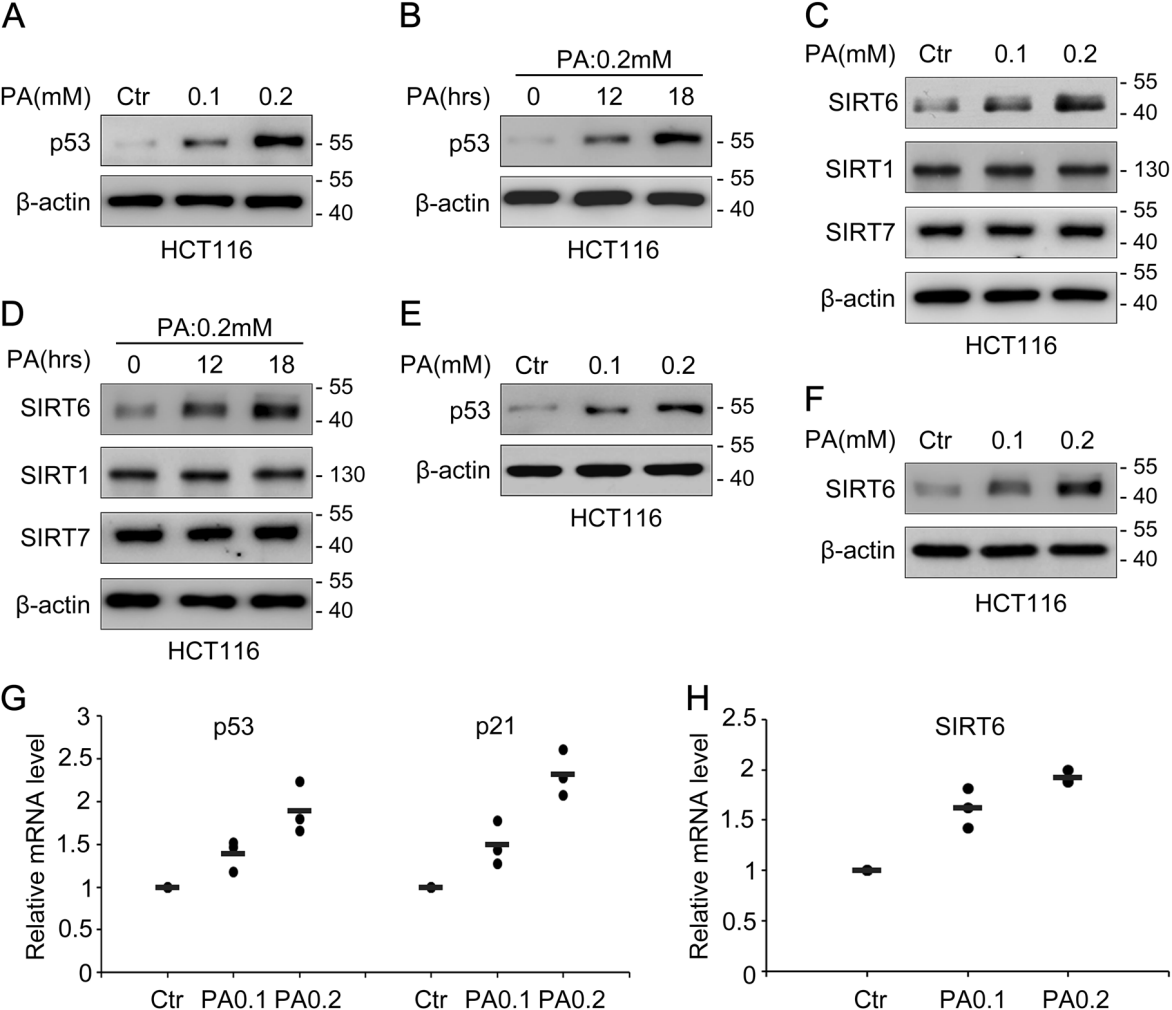
p53 and SIRT6 expression increases after palmitic acid (PA) treatment **a** HCT116 cells were treated with PA (0.1 mM and 0.2 mM) or untreated (0 mM, Ctr) for 18 h and total p53 protein expression was detected by western blotting. β-actin was used as a loading control. **b** HCT116 cells were treated with 0.2 mM PA for 0, 12 and 18 h and the total p53 protein expression was detected by western blotting. **c** HCT116 cells were treated with various doses of PA and the protein expression of total SIRT1, SIRT6 and SIRT7 was detected by Western blotting. **d** HCT116 cells were treated with 0.2 mM PA for 0, 12 and 18 h, and total SIRT1, SIRT6 and SIRT7 expression was detected by western blotting. **e**, **f** HCT116 cells were harvested in NP-40 buffer to detect the soluble levels of p53 and SIRT6 by western blotting. **g**, **h** HCT116 cells were treated as outlined above, and *p53*, *p21* and *SIRT6* mRNA expression was analyzed by real-time PCR. mRNA levels of the control sample were set as 1, and relative mRNA levels of the experimental samples were normalized to this control. The bar (-) represents the means (*n* = 3)

Given that p53 and SIRT6 are involved in many aspects of cell metabolic regulation, we asked whether they both participate in lipid homeostasis. We treated human colon cancer HCT116 cells with PA and measured the total p53 and SIRT6 protein expression levels by Western blotting. We found that the total expression levels of both proteins significantly increased in a PA dose-dependent and time-dependent manner (Fig. 1a–d). The soluble protein expression levels also increased in a dose-dependent manner (Fig. 1e, f). *p53* and *SIRT6* mRNA expression levels markedly increased after PA treatment (Fig. 1g, h), implying that the effect of PA occurs at the transcriptional level. And the mRNA level of *p21*, which is a major transcriptional target of p53 was also increased (Fig. 1g). Similar results were detected in human colon cancer LoVo cells (Supplementary Fig. S1A-D) and human liver cancer HepG2 cells (Supplementary Fig. S2A). Interestingly, although SIRT1 and SIRT7 are predominantly found in the nucleus with SIRT6, PA could only induce SIRT6 expression (Fig. 1c, d). These results suggest that p53 and SIRT6 are functional targets of PA.

### p53 interacts with SIRT6 in vivo and in vitro following PA exposure

**Fig. 2:**
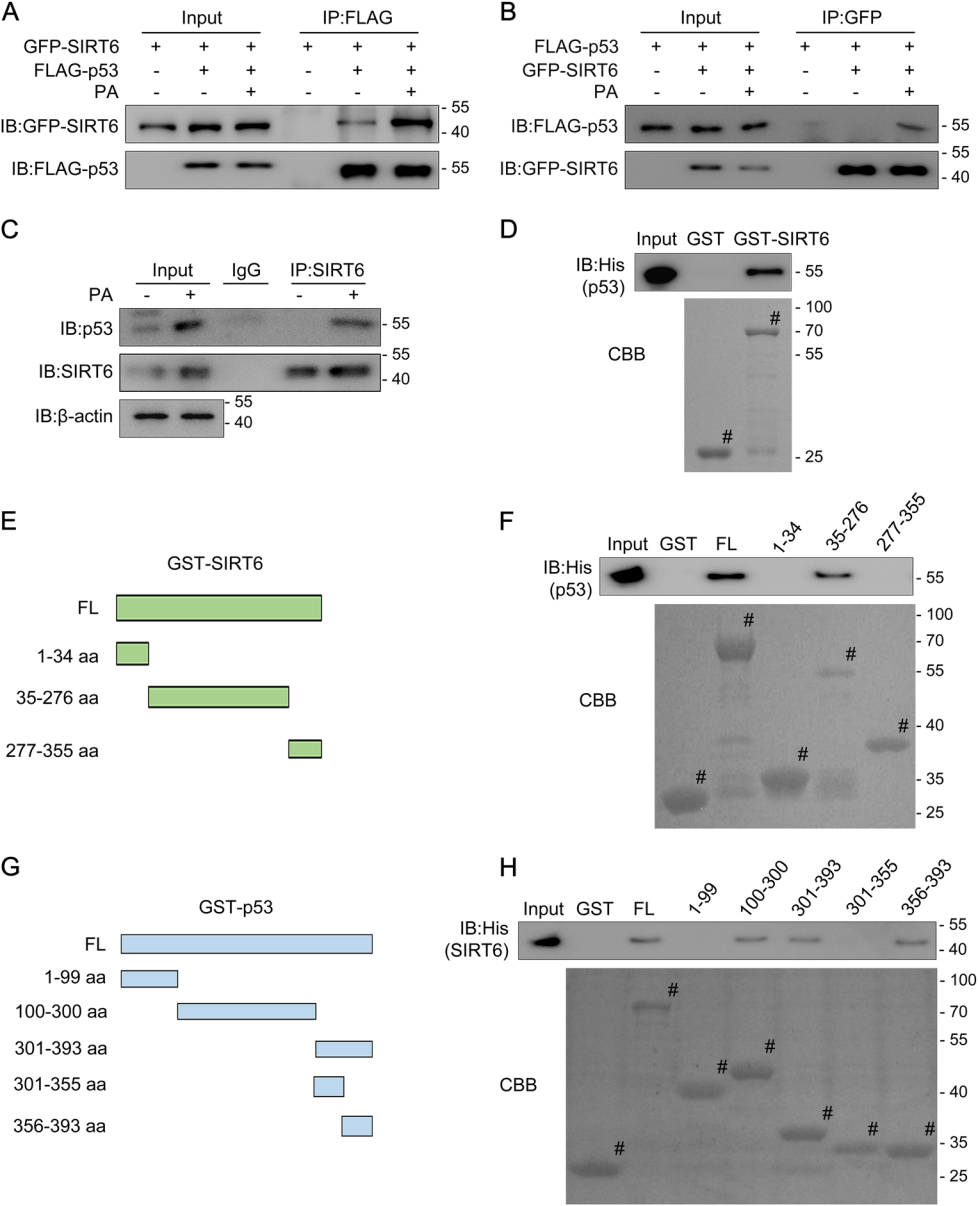
p53 interacts with SIRT6 in vivo and in vitro in response to palmitic acid (PA) treatment **a**, **b** HCT116 cells were transfected with GFP-SIRT6 and FLAG-p53 plasmids and then exposed to 0.2 mM PA for 18 h. Then, protein was extracted for co-immunoprecipitation (co-IP) using an anti-FLAG or anti-GFP antibody, followed by western blotting using an anti-GFP or anti-FLAG antibody to detect the interaction between p53 and SIRT6. **c** HCT116 cells were treated with or without 0.2 mM PA for 18 h and the protein was extracted for co-IP using an anti-SIRT6 antibody, followed by western blotting using an anti-SIRT6 or anti-p53 antibody to detect the endogenous interaction between p53 and SIRT6. **d** His-p53 protein (cloned into pET28b^+^ and expressed in bacteria) was purified in vitro and incubated with GST or a GST-SIRT6 fusion protein (purified from bacteria by using vector pGEX-4T3). Western blotting or Coomassie Brilliant Blue (CBB) staining was performed to detect the direct binding of p53 and SIRT6 in vitro. ^#^ indicates the specific bands. **e** Schematic of plasmids encoding full-length (1–355 aa, FL) SIRT6 and SIRT6 fragments (1–34 aa, N terminus; 35–276 aa, core domain; 277–355 aa, C terminus). **f** GST-SIRT6 FL or fragments were incubated with His-p53, and western blotting with an anti-His antibody or CBB staining was performed to detect the interaction. **g** Schematic of the plasmids encoding FL p53 and the p53 fragments (1–99 aa, 100–300 aa, 301–393 aa, 301–355 aa and 356–393 aa). **h** GST-p53 FL or fragments were incubated with His-SIRT6, and analyzed by western blotting with an anti-His antibody or by CBB staining to detect the interaction. IgG immunoglobulin, IB immunoblot; IP immunoprecipitation

We performed a co-immunoprecipitation (co-IP) assay to explore whether there is a molecular link between p53 and SIRT6 in response to PA. GFP-tagged SIRT6 and FLAG-tagged p53 constructs were co-transfected into HCT116 cells. The cells were then treated with PA for 18 h before lysates were collected for co-IP with either anti-GFP or anti-FLAG antibody followed by probing with anti-FLAG or anti-GFP, respectively. The interaction between the exogenous p53 and SIRT6 significantly increased after PA stimulation (Fig. 2a, b). Endogenous p53 and SIRT6 showed an enhanced interaction in HCT116 cells in response to PA treatment (Fig. 2c).

We then performed a GST pull-down assay to investigate whether the interaction between p53 and SIRT6 is direct. An His-tagged p53 protein was expressed in bacteria, purified and then incubated with GST (as a negative control) or GST-SIRT6. Here, we found that p53 directly interacts with GST-SIRT6 but not GST alone, showing that p53 can interact with SIRT6 in vitro (Fig. 2d).

To map the regions of SIRT6 involved in p53 binding, we constructed and purified full-length (FL) GST-SIRT6 (1–355 aa) and several fragments (N-terminus, 1–34 aa; core domain, 35–276 aa; and C-terminus, 277–355 aa) (Fig. 2e) and then repeated our GST pull-down assay. The FL and the SIRT6 35–276 aa fragments bound p53 (Fig. 2f), indicating that the SIRT6 core-domain is responsible for this interaction. We then reciprocally mapped the regions of p53 required for SIRT6 binding by incubating FL or fragments of GST-p53 (Fig. 2g; FL, 1–393 aa; N-terminus, 1–99 aa; DNA-binding core domain, 100–300 aa; C-terminus, 301–393 aa; the tetramerization domain, 301–355 aa; and the regulatory domain 356–393 aa) with His-SIRT6. His-SIRT6 specifically interacted with FL GST-p53, and the 100–300 aa and 356–393 fragments (Fig. 2h). These data indicate that p53 directly interacts with SIRT6 and that this interaction is enhanced in response to PA treatment.

### SIRT6 is recruited to chromatin by p53 in response to PA treatment

**Fig. 3:**
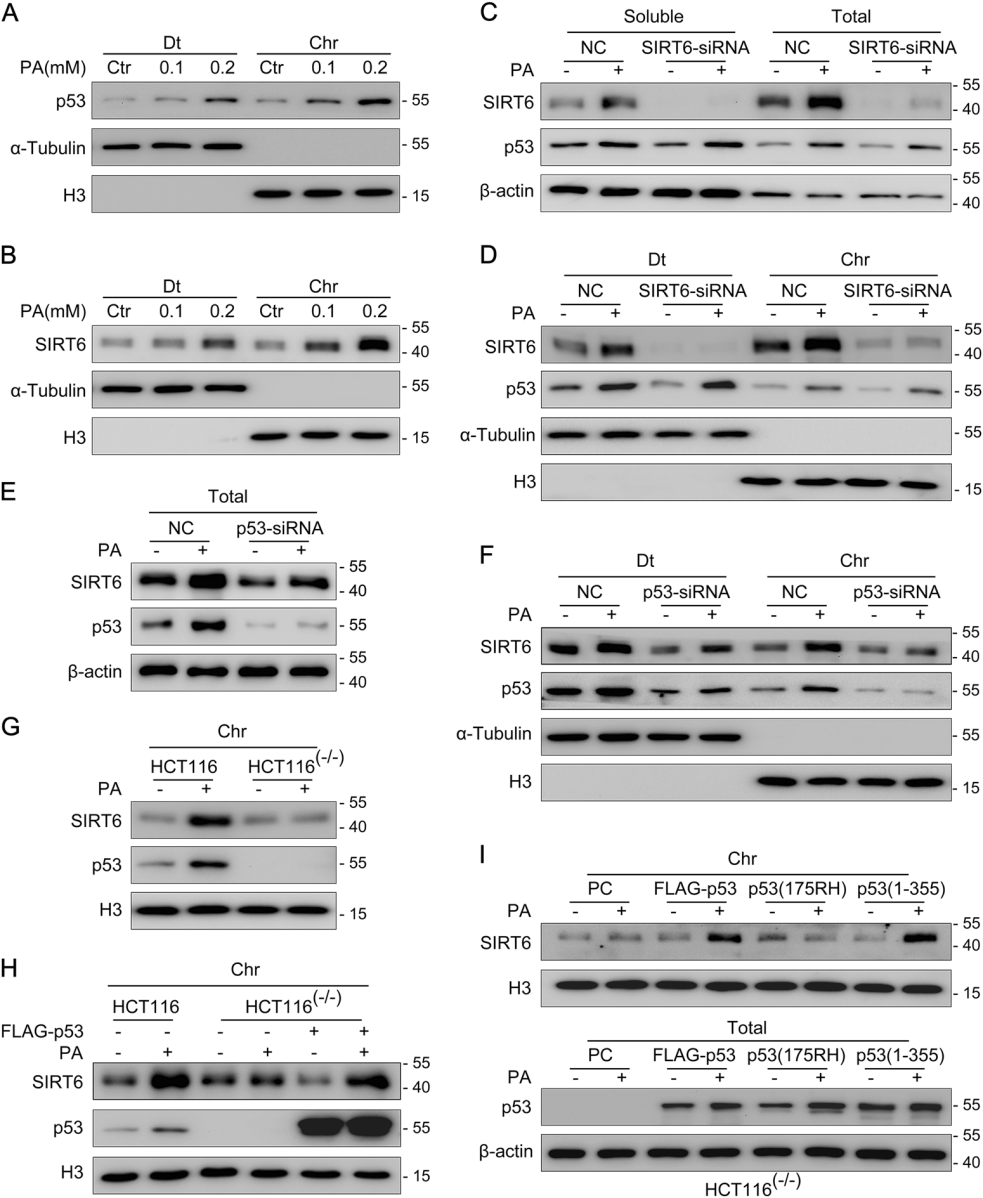
SIRT6 is recruited to chromatin by p53 in response to palmitic acid (PA) treatment **a** HCT116 cells were treated with PA (0.1 and 0.2 mM) or untreated (0 mM, Ctr) for 18 h. Detergent extractable (Dt) and chromatin (Chr) proteins were then extracted for western blotting and analyzed using a p53 antibody. α-Tubulin and histone H3 antibodies were used as the loading controls for Dt and Chr proteins, respectively. **b** Dt and Chr proteins were extracted for Western blotting and analyzed using a SIRT6 antibody. **c** SIRT6 siRNA or a non-specific siRNA negative control (NC) was delivered into HCT116 cells. The expression of soluble and total p53 and SIRT6 was detected by western blotting. **d** HCT116 cells were treated as outlined above, and then Dt and Chr proteins were then extracted for western blotting and analyzed using p53 or SIRT6 antibodies. **e** A p53 siRNA or a non-specific siRNA NC was delivered into HCT116 cells and total p53 and SIRT6 expression was detected by western blotting. **f** A p53 siRNA or a non-specific siRNA NC was delivered into HCT116 cells and Dt and Chr proteins were extracted and analyzed using antibodies against p53 and SIRT6. **g** HCT116 and HCT116 (p53^−/−^) cells were treated with PA. Chr protein was then extracted for western blotting and analyzed using antibodies against p53 and SIRT6. **h** HCT116 (p53^−/−^) cells were transfected with an empty plasmid or a FLAG-p53 and then HCT116 and HCT116 (p53^−/−^) cells were treated with PA as outlined above. Chr proteins were extracted for western blotting and analyzed using antibodies against p53 and SIRT6. **i** HCT116 (p53^−/−^) cells were transfected with an empty plasmid (PC), a full-length FLAG-p53 plasmid, a plasmid expressing p53 (175RH) in which p53 is unable to bind DNA, or a plasmid expressing p53 (1–355 aa). Total proteins and Chr proteins were extracted for Western blotting and analyzed using antibodies against p53 and SIRT6

We next monitored the localization of p53 and SIRT6, to understand the biological function of the p53 and SIRT6 interaction following PA exposure. The detergent extractable (Dt) and chromatin (Chr)-bound proteins were extracted separately, and then analyzed for p53 and SIRT6 levels. Here, we found that the expression levels of both p53 and SIRT6 were significantly increased in both the Dt and Chr fractions following PA treatment, in a PA dose-dependent manner (Fig. 3a, b). These data suggest that both p53 and SIRT6 localize to and are increased on chromatin after PA treatment.

To clarify the relationship between p53 and SIRT6 after PA treatment in HCT116 cells, we first explored the mediatory effects of SIRT6 on p53. We transfected double stranded SIRT6 siRNA or a non-specific siRNA into HCT116 cells and then repeated the PA treatment. The expression of total, soluble, Dt and Chr p53 after PA treatment did not markedly change in SIRT6 siRNA-treated cells compared to the non-specific siRNA-treated cells (Fig. 3c, d). These data indicate that SIRT6 has no effect on the expression of p53 or its increase on chromatin.

We then explored the reciprocal effect of p53 on SIRT6, by treating HCT116 cells with a p53 siRNA or a non-specific siRNA, with or without PA treatment. The total protein levels of SIRT6 decreased in p53 siRNA-treated cells compared to non-specific siRNA-treated cells (Fig. 3e). Interestingly, increase of SIRT6 on chromatin was blocked after PA stimulation in p53 siRNA-treated cells (Fig. 3f), despite the total protein levels of SIRT6 in p53 siRNA-treated cells increasing after PA stimulation (Fig. 3e). Similar results were found in HCT116 (p53^−/−^) cells (p53 null) (Fig. 3g) and human liver cancer HepG2 cells (Supplementary Fig. S2B).

To confirm the effect of p53 on SIRT6 increase on chromatin, we transfected a FLAG-p53 plasmid into HCT116 (p53^−/−^) cells and then exposed the cells to PA. The level of SIRT6 on chromatin after PA stimulation was significantly recovered in the FLAG-p53 transfected cells (Fig. 3h). This increase was not recovered when HCT116 (p53^−/−^) cells were transfected with a p53 (175RH) (arginine to histidine) plasmid, which lacks the ability to bind DNA (Fig. 3i). However, the p53 (1–355 aa) fragment could still induce SIRT6 level on chromatin, indicating that the p53 DNA-binding domain (100–300 aa) instead of the C-terminal regulatory domain (356–393 aa) is responsible for SIRT6 recruitment. These data demonstrate that p53 helps recruit SIRT6 onto chromatin via its DNA-binding activity.

### p53 and SIRT6 affect the expression of CL de novo biosynthesis-related genes after PA treatment

**Fig. 4:**
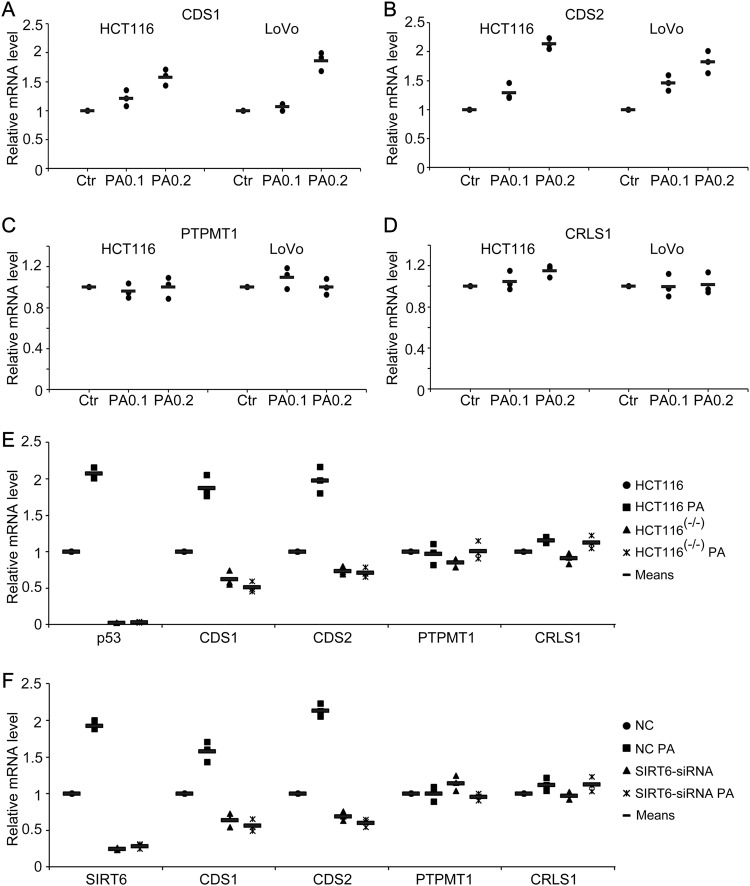
p53 and SIRT6 affect the expression of cardiolipin de novo biosynthesis-related genes in response to palmitic acid (PA) treatment **a**–**d** HCT116 and LoVo cells were treated with PA (0.1 and 0.2 mM) or untreated (0 mM, Ctr) for 18 h, and the mRNA expression of (**a**) *CDS1*, (**b**) *CDS2*, (**c**) *PTPMT1* and (**d**) *CRLS1* was analyzed by real-time PCR. **e** HCT116 and HCT116 (p53^−/−^) cells were treated as outlined above and the mRNA expression of *p53*, *CDS1*, *CDS2*, *PTPMT1* and *CRLS1* was analyzed by real-time PCR. **f** HCT116 cells were transfected with SIRT6 siRNA or a non-specific siRNA (NC) and then treated with PA for 18 h. The mRNA expression of *SIRT6*, *CDS1*, *CDS2*, *PTPMT1* and *CRLS1* was analyzed by real-time PCR. mRNA levels of the Ctr, HCT116 or NC samples were set as 1, and relative mRNA levels of the other samples were normalized to these controls. The bar (−) represents the means (*n* = 3)

To explore the role of the p53/SIRT6 axis in CL de novo biosynthesis, HCT116 cells were treated with various doses of PA (0, 0.1 mM and 0.2 mM). After 18 h, mRNA was extracted and real-time PCR was performed to analyze the expression of CL de novo biosynthesis-related genes. *CDS1* and *CDS2* expression increased in a dose-dependent manner after PA treatment in HCT116 cells (Fig. 4a, b), whereas *PTPMT1* and *CRLS1* expression was not significantly affected (Fig. 4c, d). Similar results were found in LoVo cells (Fig. 4a–d) and human liver cancer HepG2 cells (Supplementary Fig. S2C). These data indicate that PA can induce expression of CL de novo biosynthesis-related genes.

Next, we investigated the function of p53 in regulating the mRNA expression of CL de novo biosynthesis-related genes. Interestingly, *CDS1* and *CDS2* expression did not increase in HCT116 (p53^−/−^) cells after PA treatment (Fig. 4e), whereas *PTPMT1* and *CRLS1* gene expression were equivalent between HCT116 and HCT116 (p53^−/−^) cells (Fig. 4e). Finally, we explored the role of SIRT6 in this process. Increased *CDS1* and *CDS2* expression in response to PA treatment was suppressed in PA-stimulated SIRT6 siRNA-treated cells compared to non-specific siRNA-treated cells, showing that *CDS1* and *CDS2* expression is also regulated by SIRT6 (Fig. 4f). Therefore, we conclude that p53 and SIRT6 have an important role in regulating PA-induced *CDS1* and *CDS2* expression.

### SIRT6 acts as a co-activator and cooperates with p53 to regulate the expression of CL de novo biosynthesis-related genes

**Fig. 5:**
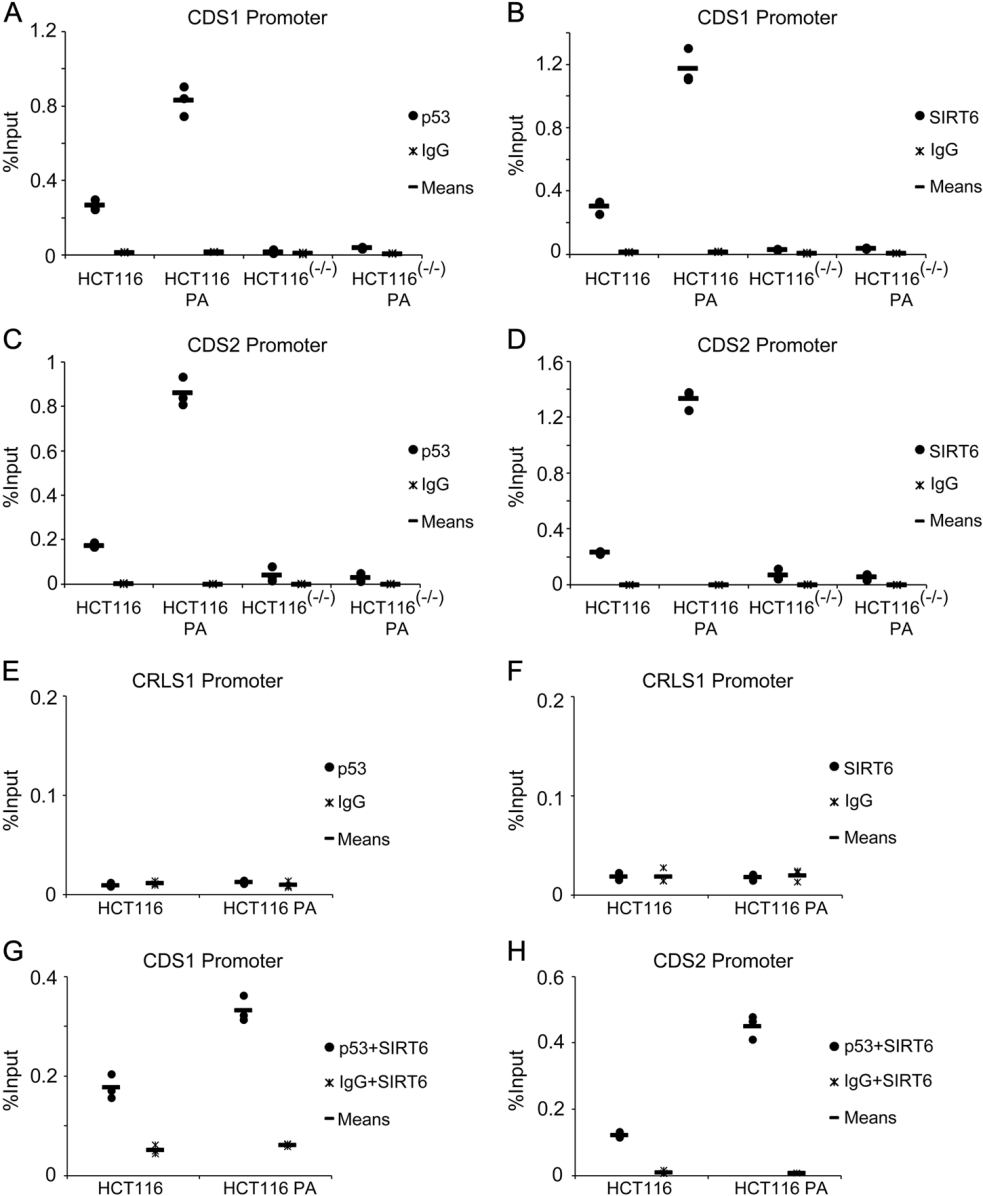
p53 recruits SIRT6 to the *CDS1/CDS2* promoters in response to palmitic acid (PA) treatment **a**–**d** HCT116 and HCT116 (p53^−/−^) cells were treated with or without 0.2 mM PA for 18 h and then harvested for chromatin immunopreciptiation (ChIP) assay to detect enrichment of p53 or SIRT6 at the *CDS1* and *CDS2* promoters. The bands containing anti-immunoglobulin G (IgG) served as negative controls. **e**, **f** HCT116 cells were treated with or without 0.2 mM PA for 18 h and then harvested for ChIP assay to detect the enrichment of p53 or SIRT6 around the *CRLS1* promoter. **g**, **h** HCT116 cells were treated with or without 0.2 mM PA for 18 h and then harvested for sequential ChIP assay to detect the enrichment of p53 and SIRT6 around the *CDS1/2* promoters. The bar (**−**) represents the means (*n* = 3)

We finally assessed the mechanism underlying how p53 and SIRT6 regulate the expression of CL de novo biosynthesis-related genes. We identified predicted, putative p53 binding sites in the *CDS1* and *CDS2* promoters from the JASPAR database, and designed real-time PCR primers around these sites. We then detected p53 localization to the *CDS1* promoter in HCT116 cells by chromatin immunoprecipitation, and found that its binding activity significantly increased following PA treatment (Fig. 5a). Next, we explored whether SIRT6 binding to the *CDS1* promoter was mediated by p53. Here we found that PA-stimulated SIRT6 binding to the *CDS1* promoter was almost completely blocked in HCT116 (p53^−/−^) cells compared to HCT116 cells (Fig. 5b). Similar results were found for the *CDS2* promoter (Fig. 5c, d). Neither p53 nor SIRT6 were detected on the *CRLS1* promoter, whose expression is not regulated by p53 or SIRT6 (Fig. 5e, f). Sequential ChIP assay revealed the co-occupancy of p53 and SIRT6 on *CDS1* and *CDS2* promoters in HCT116 (Fig. 5g, h) and HepG2 (supplementary Fig. S2D, E) cells. These data indicate that SIRT6 does not drive *CDS1* and *CDS2* gene expression when p53 is absent. Instead, p53 cooperates with SIRT6 to regulate *CDS1* and *CDS2* gene expression by recruiting SIRT6 to the promoters to further mediate CL de novo biosynthesis.

As SIRT6 is a histone deacetylase, we investigated H3K56Ac and H3K9Ac on the CDS1 and CDS2 promoters, and found H3K56Ac level was mediated by SIRT6 on their promoters after PA treatment (Supplementary Fig. S3A, B).

**Fig. 6:**
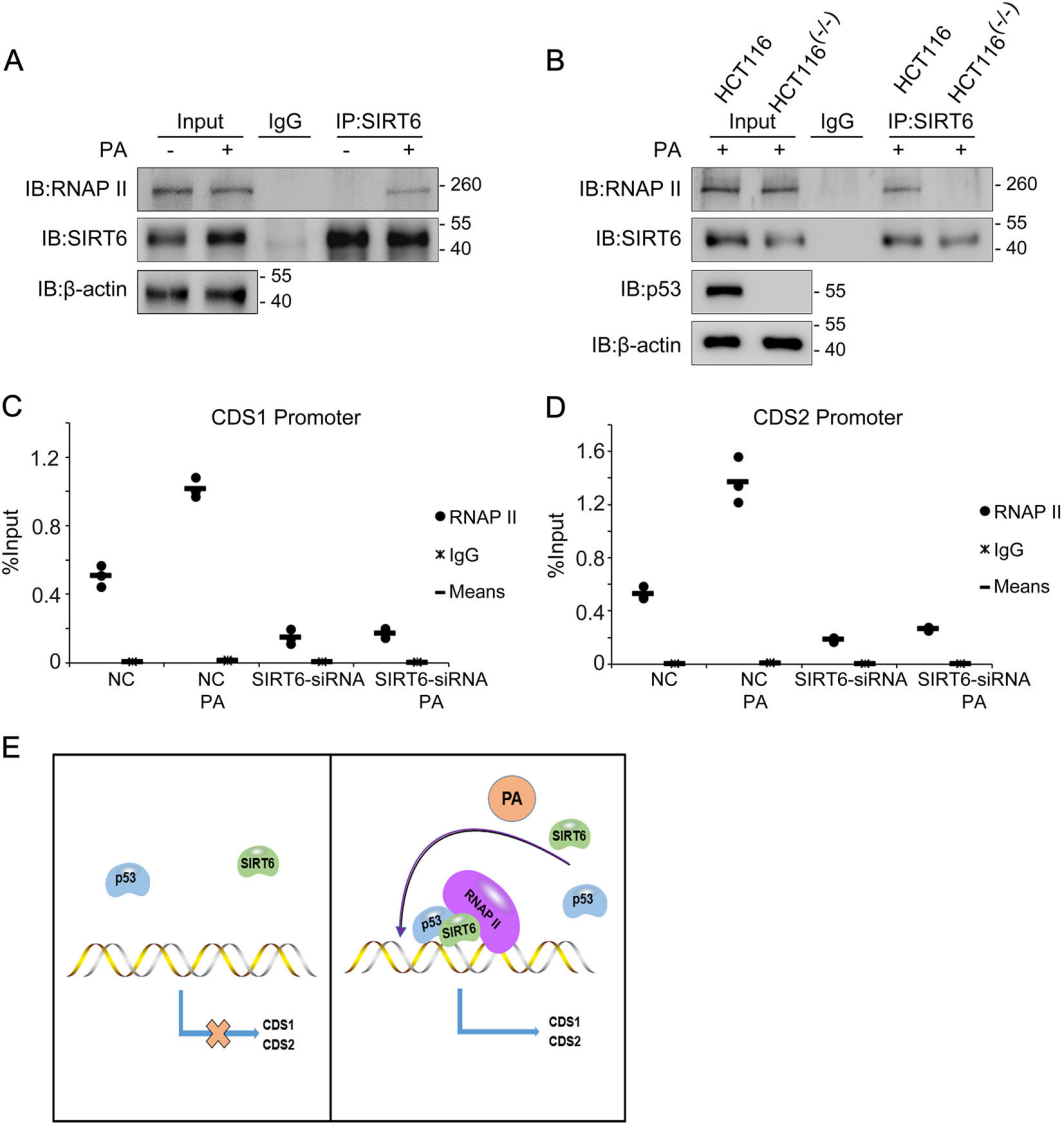
SIRT6 is a p53 co-activator and recruits RNA polymerase II (RNAP II) to the *CDS1* and *CDS2* promoters in response to palmitic acid (PA) treatment **a** HCT116 cells were treated with or without 0.2 mM PA for 18 h and proteins were extracted for co-immunoprecipitation with an anti-SIRT6 antibody, followed by western blotting with an anti-SIRT6 or anti-RNAP II antibody to detect the endogenous interaction between RNAP II and SIRT6. **b** HCT116 and HCT116 (p53^−/−^) cells were treated with 0.2 mM PA for 18 h and proteins were extracted for co-immunoprecipitation with an anti-SIRT6 antibody, followed by western blotting with an anti-SIRT6 or anti-RNAP II antibody to detect the endogenous interaction between RNAP II and SIRT6. **c**, **d** HCT116 cells were transfected with a SIRT6 siRNA or a non-specific siRNA negative control (NC) and then harvested for a ChIP assay to detect enrichment of RNAP II around the *CDS1* and *CDS2* promoters. The bands containing anti-immunoglobulin G (IgG) served as negative controls. The bar (**−**) represents the means (*n* = 3). **e** A schematic showing a possible mechanism by which p53 coordinates with SIRT6 to regulate the expression of cardiolipin de novo biosynthesis-related genes. In the presence of PA, p53 recruits SIRT6 to bind chromatin in a direct complex. Here, SIRT6 recruits RNA polymerase II to the *CDS1* and *CDS2* promoters to activate their transcription and permit CL biosynthesis. IB immunoblot, IP immunoprecipitation

Our final assays studied how SIRT6 cooperates with p53 to regulate *CDS1* and *CDS2* expression. By screening the possible regulatory factors, we identified that SIRT6 could interact with RNA polymerase II (RNAP II) following PA treatment (Fig. 6a); this interaction was disrupted in HCT116 (p53^−/−^) cells (Fig. 6b). We then investigated whether SIRT6 could recruit RNAP II to the *CDS1* and *CDS2* promoters after PA treatment. Here we found that RNAP II increases binding on the promoters of *CDS1* and *CDS2* after PA treatment, but this increase was prevented in SIRT6 siRNA-treated cells (Fig. 6c, d). In addition, mRNA of *CDS1* and *CDS2* are relatively low in SIRT6 RNAi treated cells (Fig. 4f). These data suggest that SIRT6 may serve as a co-activator of p53 to regulate CL de novo biosynthesis in the context of unbalance of lipid metabolism.

## Discussion

The present study has identified p53 and SIRT6 as new regulators of CL de novo biosynthesis, thus adding a new layer of knowledge to p53 and SIRT6 biology. In response to PA treatment, p53 and SIRT6 mRNA and protein levels increase. p53 subsequently recruits SIRT6 to chromatin, where p53 and SIRT6 bind the *CDS1* and *CDS2* promoters. Here, SIRT6 recruits RNA polymerase II to the *CDS1* and *CDS2* promoters to upregulate the expression of these enzymes that are key for CL de novo biosynthesis. As such, we propose a model, whereby SIRT6 serves as a co-activator of p53 to regulate CL de novo biosynthesis (Fig. 6e).

The link between p53 and SIRT6 was originally based on the finding of low SIRT6 protein levels in several organs of p53-deficient (p53^−/−^) mice compared to wild-type mice^32^. Mechanistically, our previous study showed that the human *SIRT6* promoter contains a predicted p53 binding site and indeed, p53 could directly bind the *SIRT6* promoter and activate its expression to regulate gluconeogenesis^30^. In addition, p53 restores SIRT6 levels after Aβ42-induced DNA damage, suggesting a mechanism of p53-dependent SIRT6 DNA damage protection in this context^33^. Furthermore, two recent studies showed that SIRT6 deacetylates p53 at lysine 382^34^ and lysine 381^31^. The former modification is sensitive to trichostatin A (TSA) inhibition^34^ and the latter negatively regulates p53 stability and activity and affects the aging process in mice^31^. Consistent with these studies, we show here a link between p53 and SIRT6 in the regulation of lipid metabolism. Our data clearly support that both p53 and SIRT6 mRNA and protein levels increase in response to PA treatment, and that p53 cooperates with SIRT6 to upregulate the CL de novo biosynthesis-related genes *CDS1* and *CDS2*. These data indicate that p53 and SIRT6 may protect against PA-induced metabolic disorders and have important roles in maintaining lipid homeostasis.

The lncRNA *LncPRESS1* has a regulatory role in SIRT6 chromatin localization. *LncPRESS1* is highly expressed in pluripotent human embryonic stem cells (hESCs) and is repressed during differentiation. *LncPRESS1* physically interacts with SIRT6 to prevent its chromatin localization, to regulate H3K56/K9Ac levels of pluripotency genes and to safeguard embryonic stem-cell status. p53 represses *lncPRESS1* expression to indirectly affect SIRT6 chromatin localization^35^. Here, however, we show that a direct interaction between p53 and SIRT6 results in SIRT6 recruitment to chromatin via the p53 DNA-binding domain (p53 100–300 aa).

As a co-activator of p53, SIRT6 enhances p53 activity in regulating *CDS1* and *CDS2* gene expression. It is already known that p53 needs a co-activator to help regulate gene expression during many physiological processes. For example, the DEAD-box helicase 5 (p68), heterogeneous nuclear ribonucleoprotein K (hnRNP K), and Src associated substrate during mitosis of 68 kDa (Sam68) co-activate p53 in the DNA damage response^36–38^. Although SIRT6 has a critical role in regulating lipid and glucose metabolism, it functions in an enzymatic-dependent manner by deacetylating specific molecules to increase or decrease their activity. In addition, SIRT6 also functions as a transcriptional repressor, using histone deacetylation to regulate various signaling pathways^39–41^. SIRT6 transcriptional activation is less reported than p53, but one study has shown that SIRT6 can interact with and recruit RNAP II to co-activate nuclear factor erythroid 2-related factor 2 (NRF2) in human mesenchymal stem cells, and deacetylation of H3K56 by SIRT6 accounts for recruitment of RNAP II^42^. In this study, we found H3K56Ac level was mediated by SIRT6 on *CDS1* and *CDS2* promoters after PA treatment (Supplementary Fig. S3A, B). Consistently, we provide evidence that SIRT6 serves as a co-activator to help p53 regulate lipid metabolism by recruiting RNAP II to the *CDS1* and *CDS2* promoters.

Taken together, we have identified a novel function of p53 and SIRT6 in CL de novo biosynthesis. Understanding this new role of p53 and SIRT6 will be useful for the future design of effective therapeutic targets to help regulate lipid homeostasis or treat metabolic diseases.

## Materials and methods

### Cell culture and palmitic acid (PA) treatment

Human colon cancer cell lines HCT116, HCT116 (p53^−/−^), LoVo, and human liver cancer cell line HepG2 were purchased from the American Type Culture Collection (ATCC). HCT116 and HCT116 (p53^−/−^) cells were cultured in McCoy’s 5a medium supplemented with 10% heat-inactivated fetal bovine serum (FBS). LoVo cells were cultured in F-12K medium supplemented with 10% heat-inactivated FBS. HepG2 cells were cultured in DMEM medium supplemented with 10% heat-inactivated FBS. All the cells were grown in the presence of penicillin/streptomycin in a 37 °C incubator with a humidified, 5% CO_2_ atmosphere. PA was purchased from Sigma (St Louis, MO, USA) and prepared at a stock concentration of 100 mM and stored at room temperature. For PA treatment, the PA stock solution was freshly added to the medium at various doses, and then incubated at 37 °C for the indicated time intervals. Control cells were treated with a control solution without PA at equivalent exposure times.

### Protein extraction and western blotting

To extract chromatin proteins, cells were lysed on ice in buffer I (150 mM NaCl, 50 mM Hepes, pH 7.5, 1 mM EDTA), with 0.1% Triton X-100 and a protease inhibitor cocktail (Roche) for 3 min. After centrifugation at 13,000 × *g* for 3 min, the detergent extractable (Dt) supernatant was collected. The cell pellets were then washed twice in PBS and lysed in buffer II (150 mM NaCl, 50 mM Hepes pH 7.5, 1 mM EDTA, 200 μg/ml RNaseA) with a protease inhibitor cocktail for 30 min at 25 °C with gentle agitation. After centrifugation at 12,000 *g* for 3 min, the remaining pellets (chromatin protein) were re-suspended in 2X SDS loading buffer, boiled, and sonicated for solubilization. The total and soluble proteins were also extracted and protein expression was detected by Western blotting as previously described with minor modifications^43^. Equal amounts of proteins were size fractionated by 9 to 15% sodium dodecyl sulfate (SDS)-polyacrylamide gel electrophoresis. Antibodies used included: anti-SIRT6 (2590 s, Cell Signaling, Danvers, MA, USA), anti-H3 (ab1791, abcam, Cambridge, MA, USA), anti-SIRT1 (sc-74465, Santa Cruz, CA, USA), anti-SIRT7 (sc-135055, Santa Cruz), anti-p53 (sc-126, Santa Cruz), anti-RNAP II (ab817, abcam), anti-β-actin (TA-09, Zsbio, Beijing, China), anti-tubulin (BE0031, EASYBIO, Beijing, China), anti-Flag (F1804, Sigma), anti-glutathione S-transferase (C1303, Applygen, Beijing, China), anti-green fluorescent protein (GFP) (D153–3, MBL, Naka-ku, Nagoya, Japan), and anti-His (PM032, MBL).

### RNA extraction and real-time PCR

Total RNA was isolated using TRIzol reagent (TianGen, Beijing, China). cDNA was synthesized from 2 μg RNA using a Quantscript RT Kit (Promega, WI, USA), according to the manufacturer’s instructions. The primer sequences used for real-time PCR were as follows: Actin-F, 5′-CCAACCGCGAGAAGATGA-3′, Actin-R, 5′-CCAGAGGCGTACAGGGATAG-3′; p53-F: 5′-GCTCGACGCTAGGATCTGAC-3′, p53-R: 5′-GCTTTCCACGACGGTGAC-3′; SIRT6-F: 5′-CTTGGCACATTCTTCCACAA-3′, SIRT6-R: 5′-GCTTCCTGGTCAGCCAGA-3′; CDS1-F: 5′-GTGTTTGGATTCATTGCTGCCT-3′, CDS1-R: 5′-AGGGCTCACATTCTGTCACG-3′; CDS2-F: 5′-GATACCCCGGAGGTCCTCAAT-3′, CDS2-R: 5′-ATCTGAACGCACATCACGATT-3′; PTPMT1-F: 5′-CAGAGGAGGCTGTAAGAGCCA-3′, PTPMT1-R: 5′-TGTGGATGTATGACCGGATCT-3′; CRLS1-F: 5′-TTGTCAATGACGAGAATTGGCT-3′, CRLS1-R: 5′-GCCCAGTTTCGAGCAATAAATCC-3′; p21-F: 5′-TGTCCGTCAGAACCCATGC-3′, p21-R: 5′-AAAGTCGAAGTTCCATCGCTC-3′.

### RNA interference (RNAi)

RNAi was performed as previously described^44^. The sequences of the SIRT6, p53 and nonspecific RNAi oligonucleotides were as follows: SIRT6 siRNA, 5′-AAGAATGTGCCAAGTGTAAGA-3′; p53 siRNA, 5′-CUACUUCCUGAAAACAACG-3′; non-specific siRNA, 5′-UUCUCCGAACGUGUCACGU-3′. These RNAi oligonucleotides and negative controls (non-specific siRNA) were transfected into HCT116 cells using a Lipofectamine 2000 transfection kit (Invitrogen, Carlsbad, CA, USA), according to the manufacturer’s instructions. Cells were harvested 48 h after transfection and subjected to Western blotting or chromatin immunoprecipitation (ChIP).

### GST pull-down assay

GST or GST fusion proteins were expressed in bacteria induced with isopropyl-β-D-thio-galactoside and purified with glutathione-Sepharose 4B beads (GE Healthcare, Little Chalfont, UK). Recombinant His-tagged proteins were purified from bacteria by Ni (ii)-Sepharose affinity (GE Healthcare). His-tagged proteins were incubated with GST fusion proteins in TEN buffer (10 mM Tris-HCl, pH 8.0, 1 mM EDTA, 100 mM NaCl) for 4 h at 4 °C. The beads were washed three times with TEN buffer and then boiled in 2X SDS loading buffer. Proteins were analyzed by Western blotting with anti-GST or anti-His antibody and by Coomassie brilliant blue staining.

### Co-immunoprecipitation (co-IP)

After treatment, HCT116 cells were harvested and lysed in lysis buffer. Antibodies were then added to the supernatant and incubated on ice for 1 h. Agarose G was then added, and the samples were mixed by rolling at 4 °C for 1 h. The beads were washed three times with lysis buffer, and the pellets were dissolved in 2X SDS loading buffer after centrifugation. The proteins were analyzed by Western blotting with the indicated antibodies.

### Chromatin immunoprecipitation (ChIP) and sequential-ChIP

HCT116 cells were cross-linked with 1% formaldehyde for 10 min at 37 °C and then washed with cold PBS. The cell pellet was resuspended in lysis buffer and then sonicated to produce an average DNA length of 500–1000 bp. The indicated antibodies were added to each sample, and the samples were mixed by rotation at 4 °C overnight. Protein A/G Sepharose beads were added to the complexes and incubated for 2 h at 4 °C. The agarose was washed sequentially with low-salt, high-salt, LiCl, and TE buffer, and eluted with elution buffer (1% SDS and 0.1 M NaHCO_3_). The cross-link was reversed at 65 °C overnight, and the DNA was dissolved in Tris-EDTA buffer and analyzed by real-time PCR.

For sequential ChIP, the primary immunoprecipitated complexes were eluted in buffer (10 mM DTT, 500 mM NaCl and 0.1% SDS) at 37 °C instead of elution buffer (1% SDS and 0.1 M NaHCO_3_). Samples were diluted 1:50 in dilution buffer (1% Triton X-100, 2 mM EDTA, 150 mM NaCl, and 20 mM Tris-HCl at pH 8.1) and immunoprecipitated with the second antibody. The anti-p53 antibody was used for the first IP and the anti-SIRT6 antibody was used for the second IP. A non-specific IgG antibody was used as a control.

The primer sequences used for ChIP real-time PCR were as follows: CDS1 promoter sequence (−301 to −180) CDS1-F: 5′-CCTGCAAGTTCTCAACGCTC-3′, CDS1-R: 5′-CTGCGTGCTGGCAGGT-3′; CDS2 promoter sequence (−1491 to −1223) CDS2-F: 5′-CCCCAAAACTCACAGCTCAG-3′, CDS2-R: 5′-ACACTTGCACACAAAAGAATTGC-3′; CRLS1 promoter sequence (−352 to −196) CRLS1-F: 5′-AACCACCCACAAGAACCGT-3′, CRLS1-R: 5′-GGGAGAAACGAACAAGATAGGA-3′.

## Electronic supplementary material

Supplementary figure legends

SUPPLEMENTAL figure 1

SUPPLEMENTAL figure 2

SUPPLEMENTAL figure 3
