# Supplementary material for: p53 cooperates with SIRT6 to regulate cardiolipin de novo biosynthesis
Source: Cell Death Dis. 2018 Sep 20;9(10):941. doi: 10.1038/s41419-018-0984-0 (PMC6148051; doi:10.1038/s41419-018-0984-0)
Supplement: Supplementary file 1 — Supplementary figure legends [file 41419_2018_984_MOESM1_ESM.docx]

**Supplementary figure legends**

**Supplementary Figure 1. p53 and SIRT6 expression increases after palmitic acid (PA) treatment in LoVo cells**

**A.** LoVo cells were treated with PA (0.1 mM and 0.2 mM) or untreated (0 mM, Ctr) for 18 h and the total protein was extracted. Western blotting was performed to detect total p53 and SIRT6 protein expression. β-actin was used as a loading control. **B.** LoVo cells were harvested in NP-40 buffer for Western blotting to detect soluble p53 and SIRT6 protein expression. **C.** LoVo cells were treated as outlined in (A), and *p53* mRNA expression was analyzed by real-time PCR. **D.** LoVo cells were treated as outlined in (A), and *SIRT6* mRNA expression was analyzed by real-time PCR. mRNA levels of the control sample were set as 1, and relative mRNA levels of the experimental samples were normalized to this control. The bar (**-**) represents the means (n=3).

**Supplementary Figure 2.** **Effect of p53 and SIRT6 on *CDS1* and *CDS2* expression after palmitic acid (PA) treatment in HepG2 cells**

**A.** HepG2 cells were treated with PA (0.1 mM and 0.2 mM) or untreated (0 mM, Ctr) for 18 h, and *p53* and *SIRT6* mRNA expression was analyzed by real-time PCR. **B.** A p53 siRNA or a non-specific siRNA NC was delivered into HepG2 cells and Chr proteins were extracted and analyzed using antibodies against p53 and SIRT6. Histone H3 antibodies were used as a loading control for Chr proteins. **C.** HepG2 cells were treated as outlined in (A), and *CDS1* and *CDS2* mRNA expression was analyzed by real-time PCR. **D-E.** HepG2 cells were treated with or without 0.2 mM PA for 18 h and then harvested for sequential ChIP assay to detect the enrichment of p53 and SIRT6 around the *CDS1/2* promoters. The bar (**-**) represents the means (n=3).

**Supplementary Figure 3. SIRT6 deacetylates H3K56 on the *CDS1/2* gene promoter**

**A-B.** HCT116 cells were transfected with a SIRT6 siRNA or a non-specific siRNA negative control (NC) and then harvested for a ChIP assay to detect enrichment of H3K56Ac and H3K9Ac on the *CDS1* and *CDS2* promoters. The bands containing anti-immunoglobulin G (IgG) served as negative controls. The bar (**-**) represents the means (n=3).
