# Supplementary figures and images for: p53 cooperates with SIRT6 to regulate cardiolipin de novo biosynthesis
Source: Cell Death Dis. 2018 Sep 20;9(10):941. doi: 10.1038/s41419-018-0984-0 (PMC6148051; doi:10.1038/s41419-018-0984-0)

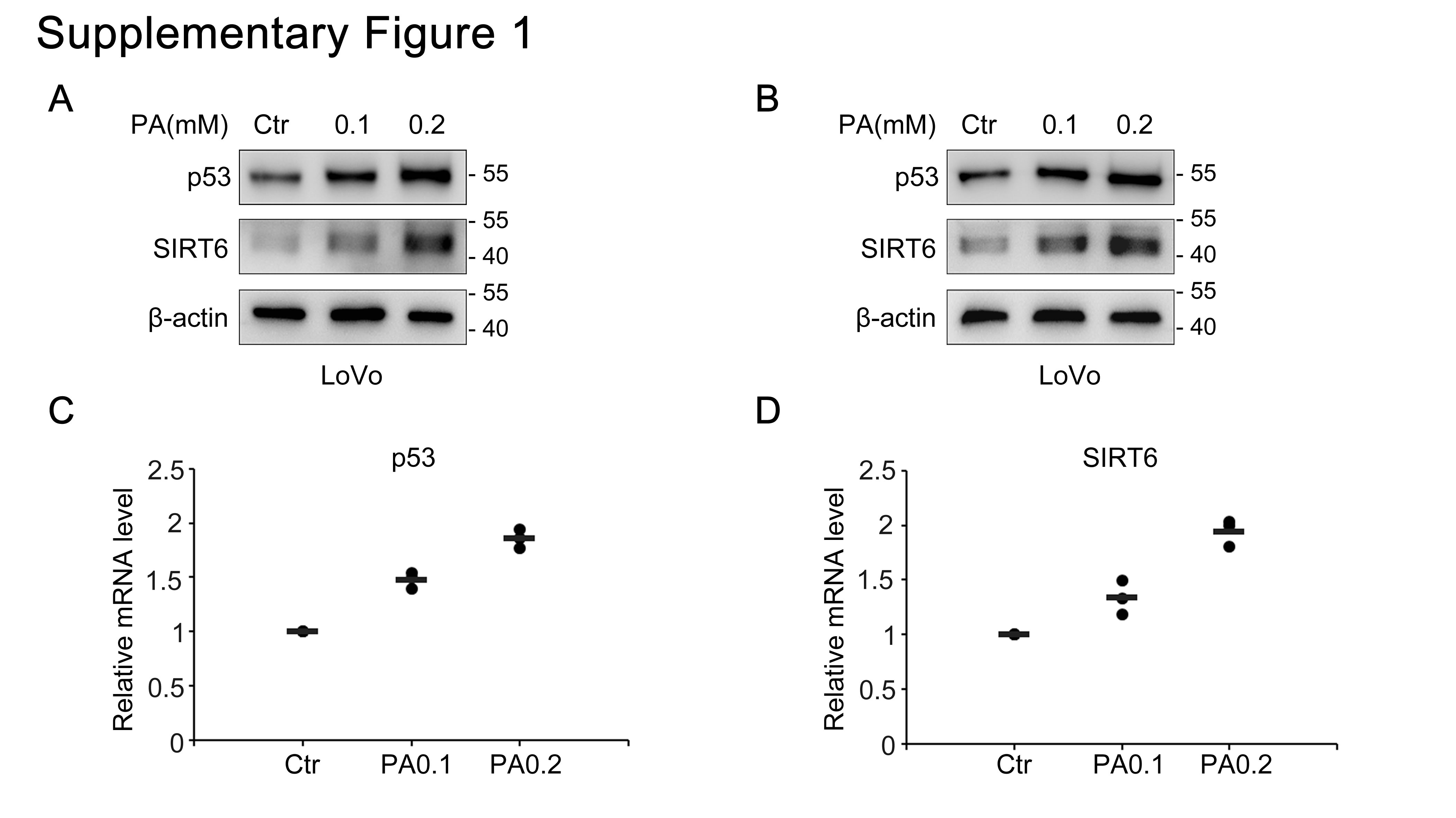

Supplement: Supplementary file 2 — SUPPLEMENTAL figure 1 [file 41419_2018_984_MOESM2_ESM.jpg]

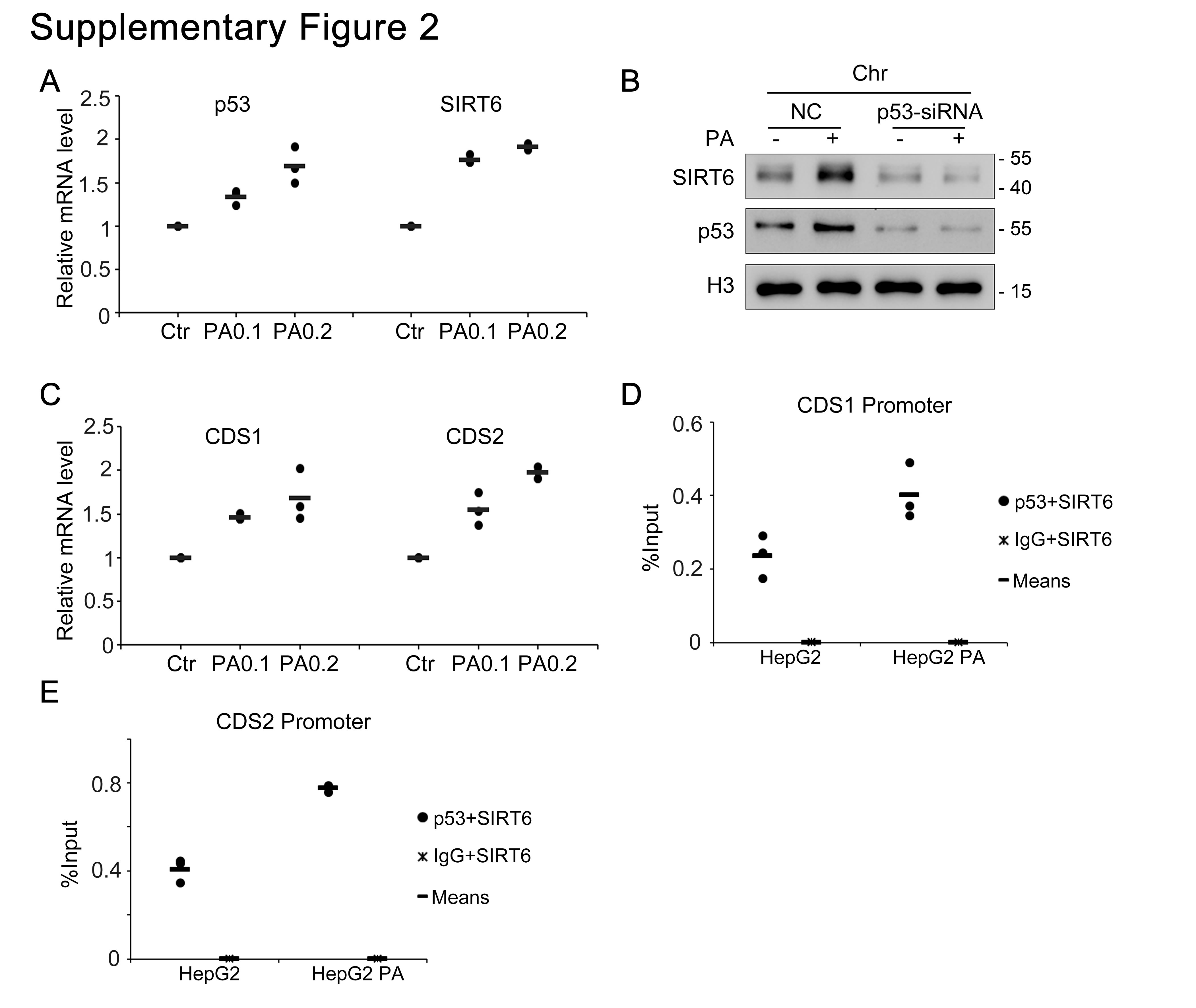

Supplement: Supplementary file 3 — SUPPLEMENTAL figure 2 [file 41419_2018_984_MOESM3_ESM.jpg]

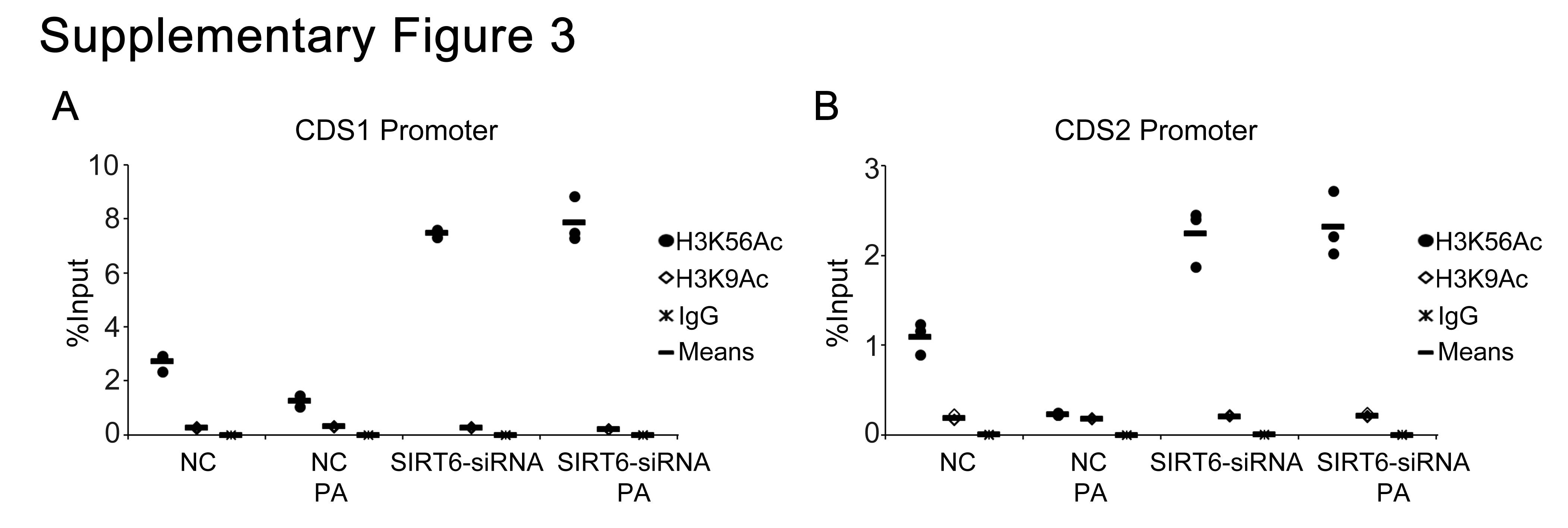

Supplement: Supplementary file 4 — SUPPLEMENTAL figure 3 [file 41419_2018_984_MOESM4_ESM.jpg]
